# Light-Triggered Bending in Photochromic/Graphene Oxide Bilayers via Synergistic Photo-Thermal Actuation and Mechanical Amplification

**DOI:** 10.1021/acsami.6c04058

**Published:** 2026-06-03

**Authors:** Lorenzo Lavista, Leonardo Vicarelli, Maria Murace, Alberto Portone, Filippo Fabbri, Federica Bianco, Stefano Roddaro, Alessandro Tredicucci, Andrea Camposeo, Luana Persano, Dario Pisignano

**Affiliations:** † Dipartimento di Fisica, 9310 Università di Pisa, Largo B. Pontecorvo 3, I-56127 Pisa, Italy; ‡ Istituto Nanoscienze-CNR, NEST-SNS, Piazza S. Silvestro 12, I-56127 Pisa, Italy

**Keywords:** photochromic molecules, reduced graphene oxide, optical control, soft actuators, bilayer devices

## Abstract

The design of soft actuators remotely controlled by optical triggering and propelling schemes requires materials that might combine ease of device fabrication and miniaturization with robust performance of moving parts and versatile and precise response to light stimuli. Here, light-triggered photochromic-doped poly­(methyl methacrylate)/reduced graphene-oxide bilayers are proposed as effective actuation architectures, showing multi-photoaddressable bending and distinctive photothermal properties. By interlayer thermomechanical contrast and intrinsic mechanical amplification of photoinduced strain mismatch, these hybrid systems show good bending and load-lifting performance, broadband photothermal triggering, as well as faster thermal and actuation response compared to pristine photochromic films. Under UV illumination, measured displacements, exerted force, and response time reach values of about 200 μm, 2 mN, and the scale of few seconds, respectively. The photothermal conversion efficiency of the system is estimated to be at least 55%. Such properties make these architectures appealing as actuation elements for various device platforms, including segments of biomimetic components, and biomedical devices with precise structure–function design of the optical control.

## Graphical abstract

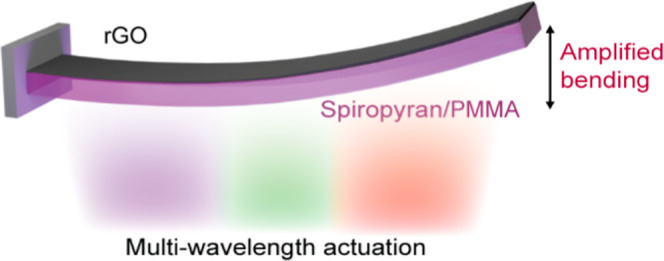

## Introduction

Light-driven, soft actuators enable remote, wireless, and spatially resolved control of moving parts. These properties make them highly appealing for various applications, including untethered microrobotics and intelligent bionics, reconfigurable optics, adaptive surfaces with intrinsic shape control, and many components and microsystems such as valves, shutters, and switches.^1–4^ At variance with electrical or pneumatic actuation techniques, optical stimuli can be delivered without physical connections and patterned with high spatial precision. They are also very versatile, since they can be tailored in their wavelength and intensity in order to encode multiple commands and functions in the same device footprint. Furthermore, avoiding the use of on-board heaters or other control components, photocontrolled architectures can be lighter and lead to microrobots with lower intrinsic cost of transport. In general, optical actuation proceeds through either photochemical routes, where molecular switching generates internal stress, or photothermal routes, where absorbed photons ultimately lead to heat production, then converted into motion through thermomechanical mismatch.^1–5^ Importantly, all these aspects can be tuned or enhanced by the incorporation of light-triggered nanodopants.

For instance, photochromic compounds provide a direct molecular handle for optical programmability, since their reversible photoisomerization can produce pronounced changes of various physical properties, such as absorption, refractive index, polarity, and local packing.^6^ Spiropyrans are a prototypical class: irradiation drives interconversion between the closed spiropyran (SP) and the open merocyanine (MC) form, accompanied by large, reversible changes of the electronic structure and of the absorption in the visible spectral range.^7,8^ In polymer hosts, this molecular photoswitch might influence not only optical density but also the local interactions of photochromic molecules with an embedding polymer matrix, namely, the mechanical response and internal stress fields. A significant milestone for actuators based on photochromic molecules was the evidence that nominally single-layer films can bend reversibly under light. Athanassiou and co-workers established SP-doped poly­(ethyl methacrylate-*co*-methyl acrylate) films as a simple, microfabrication-compatible platform for light-triggered bending.^9^ Beyond cantilever bending, SP-doped methacrylate films were also used to realize photoinduced dimensional changes in patterned structures, such as switchable diffraction gratings,^10^ and were found to show reversible, light-tunable stiffness, i.e., storage modulus changing by several percent under alternating UV/visible exposure.^11^ These effects were attributed to phototuned MC–polymer interactions and/or free-volume changes caused by MC aggregation.^11^

A different strategy to obtain large, repeatable deflection in beam microcomponents relies on an intrinsic or engineered built-in asymmetry.^2,6^ For instance, these properties can be highlighted by engineering photochromic polymer films together with a layer embedding different nanosystems that provide high thermo-mechanical contrast. Such a method can be used to easily produce elongated bending structures in the form of beams or cantilevers. In addition, these devices can show enhanced bending response since small internal strain gradients or mismatches can lead to large curvatures and edge displacements by mechanical amplification. Polymer bilayers have been recently used for the modular design of mechanically compliant, electrothermally activated worm-like robots.^12^ In photocontrolled bilayered devices, light absorption would be converted into motion using photothermal effects, with differential strain across the thickness. While SP-hydrogels were used to demonstrate light-induced swelling in bilayer structures,^13,14^ photocontrolled glassy polymer films can be advantageous in this respect since they are not affected by limiting kinetics related to slow solvent diffusion. This would largely suppress swelling and make photothermal transduction a relevant and possibly dominant mechanism. Carbon-based photothermal layers (nanotubes, graphene, graphene oxide, etc.), which efficiently convert light into heat, are highly appealing candidates to be coupled with photochromic layers. Indeed, these layers provide strong thermomechanical contrast with polymers, often exhibiting low or negative thermal expansion, and can facilitate rapid in-plane heat spreading.^15,16^ Actuators based on graphene oxide and related graphene–polymer bilayer cantilevers have been evidenced to exhibit large, low-power, and programmable deformation.^16–23^ Hence, the combination of graphene systems with photochromic polymer layers is interesting, as an architecture allowing the thermomechanical mechanisms of photochromic actuation to be better elucidated and as a potential candidate for optically controlled segments in biomimetic or biomedical components.

Here, we investigate in depth the light-triggered bending of SP-doped poly­(methyl methacrylate) (PMMA)-reduced graphene-oxide (rGO) bilayers by exploiting photothermal mechanisms to achieve controlled displacement. Benchmarking the response of these devices against freestanding single-layer SP-doped PMMA films, we find that the deflection of thick (tens of μm) cantilevers cannot be explained by a purely through-thickness photothermal gradient model. While illumination mainly provides only modest temperature triggers, fabrication-imprinted structural gradients are likely to fix the bending polarity. In addition, in the bilayered architecture, coupling high thermomechanical contrast with intrinsic mechanical amplification of photoinduced strain mismatch significantly enhances the curvature and maximum achievable edge displacement and leads to significantly shorter thermal rise and actuation times. These hybrid architectures are very interesting for the low-cost integrated design of photocontrolled systems, where functional properties of different nanomaterial components can be used for effective compliance and control.

## Results and Discussion

Our bilayer system is displayed in Figure 1. It is formed by incorporating the 1′,3′-dihydro-1′,3′,3′-trimethyl-6-nitrospiro­[2H-1-benzopyran-2,2′-(2H)-indole] (6-NO_2_–BIPS) SP derivative in a PMMA matrix (thickness 60 μm, 6-NO_2_–BIPS content 2.5 wt %), and then drop-casting a ∼15 μm thick top layer from a dispersion of rGO in ethanol (see Methods and Figure S1 in the Supporting Information for details). The photoisomerization of SP into its open merocyanine (MC) form upon UV illumination, and the reverse process obtained upon either exposure to visible light or thermal relaxation, are schematized in the left part of Figure 1. SP is nonpolar and colorless, with optical absorption at ∼350–400 nm, whereas MC is polar and shows a strong absorption peaked in the 530–600 nm range.^7,24^ Nitro-substituted SP systems are reported to show large (60–85%) quantum yields of photochromic conversion in solvents with low-polarity,^7^ decreasing to 10–20% or less in more polar environments and polymer matrices.^7,25^ Compared to other reported bilayered architectures,^16–20,23,26–28^ the photochromic dopant coupled with rGO leads to an appealing combination of lightweight construction, easy device manufacturing, and stable light-driven actuation properties, as shown in the following. The broadband optical transmission of the bilayers strongly decreases upon depositing increasing amounts of rGO (Figure S2), reaching values of a few percent at high surface density of cast rGO. The corresponding rGO surface coverage, Σ (i.e., the ratio of the area of the rGO-covered region to the total top surface area), highlights the formation of a connected network of rGO microparticles, occurring above a two-dimensional percolation threshold^29^ surface density at about 100 μg cm^–2^ (58% ± 6% average surface coverage, right part of Figure 1, and Figure 2).

**Figure 1:**
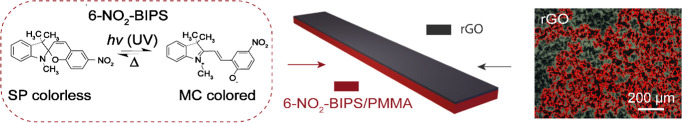
Schemes of the chemical structure and isomerization of 6-NO_2_–BIPS (left), 6-NO_2_–BIPS/PMMA-rGO as photoresponsive bilayer (middle), and rGO micrograph highlighting large percolating clusters by red outline (right, surface coverage 108 μg cm^–2^).

**Figure 2:**
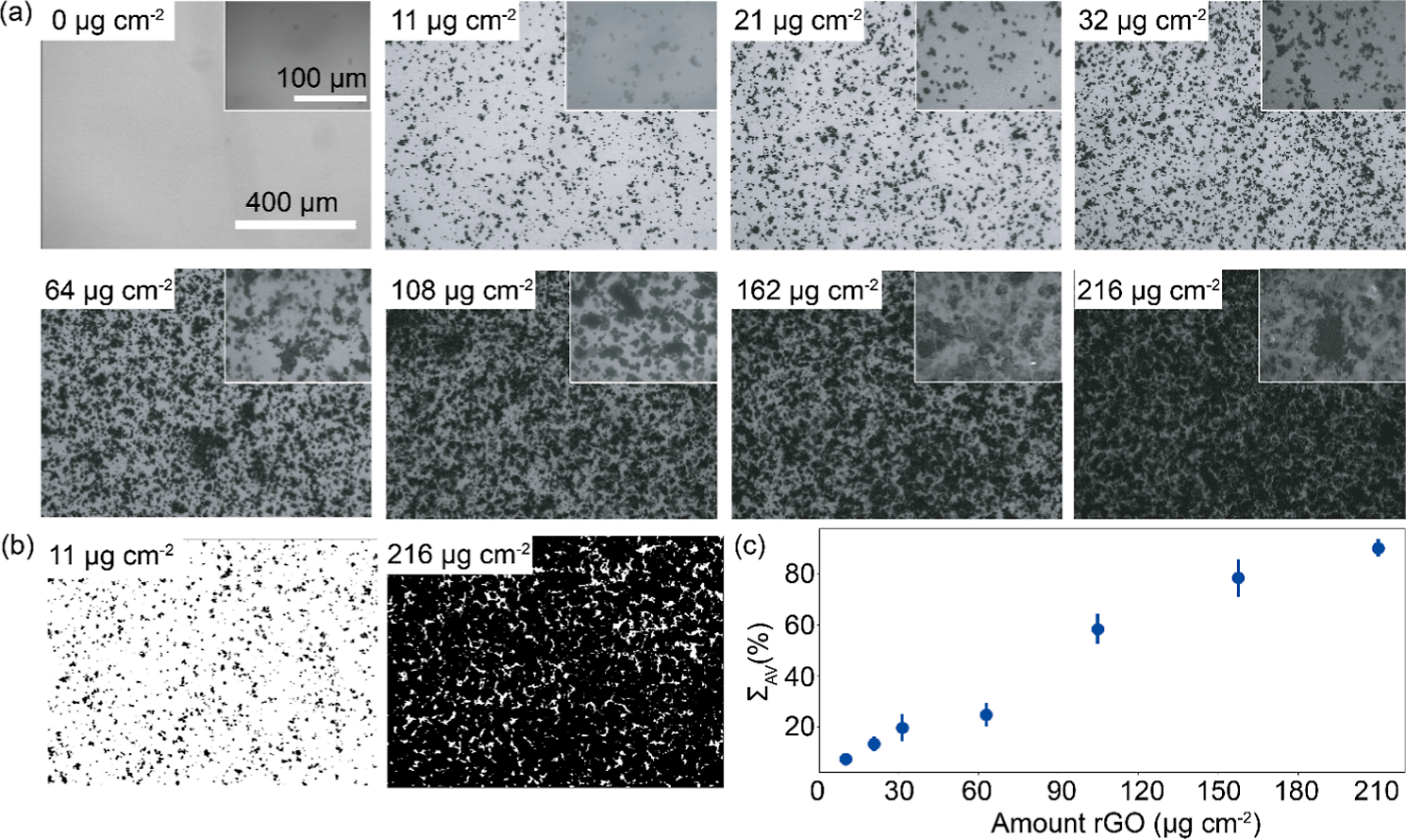
(a) Optical micrographs of samples realized by using different amounts of deposited rGO. (b) Example of processed images obtained by applying a threshold-filter. (c) Surface coverage average values, Σ_AV_, obtained by analyzing four images for each cast dispersion vs the amount of cast rGO. Σ_AV_ increases with the amount of cast rGO dispersion, with values ranging from 7.5% (at 11 μg cm^–2^) to 90% (at 216 μg cm^–2^).

Cross-sectional images of 6-NO_2_–BIPS/PMMA-rGO, collected by scanning electron microscopy (SEM, Figure S3a) and fluorescence confocal microscopy (Figure S3b), also suggest that the rGO component starts to be incorporated into the polymer layer for densities above 150 μg cm^–2^. Overall, a surface density of 108 μg cm^–2^ is found to offer both good rGO coverage and a well-defined bilayer structure. Raman mapping analysis on these samples confirms abundant presence of rGO with good reduction (Figure 3). The rGO coverage features two broad Raman peaks centered at ∼1600 (G band) and ∼1300 cm^–1^ (D band),^30^ whereas PMMA has a rich Raman spectrum with relevant bands at 940–990 (O–CH_3_ rocking), 1452 (C–H bending), and 1729 cm^–1^ (C=O stretching).^31^

**Figure 3:**
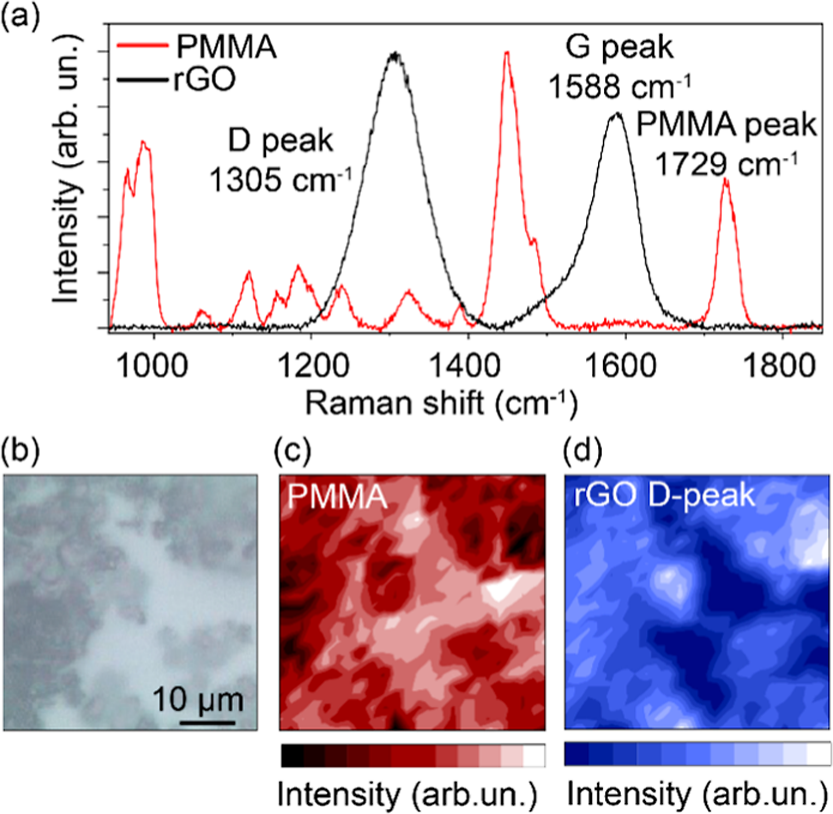
(a) Raman spectra of PMMA and rGO. The ratio between the intensities of the D and G bands (*I*_D_/*I*_G_) of rGO is between 1.4 and 1.6, suggesting good reduction.^30^ (b–d) Raman mapping of a PMMA-rGO bilayer (top view), without 6-NO_2_–BIPS (cast rGO: 108 μg cm^–2^): (b) optical image of the mapped area; (c,d) maps of the intensity of the 1729 cm^–1^ (PMMA) and the 1305 cm^–1^ (rGO D-peak), respectively. Dark red/blue in (c,d) corresponds to low peak intensity.

To evaluate the synergetic role of the photochromic-doped layer and of rGO in light-driven actuation, we realize several cantilever prototypes and compare their photoactivated displacement. First, we fabricate cantilevers either with or without rGO. Using a multicolor laser source (UV-blue at 405 nm, green at 561 nm, and red at 638 nm, all with 3.3 mW cm^–2^), we illuminate a 6-NO_2_–BIPS/PMMA and a 6-NO_2_–BIPS/PMMA-rGO cantilever with a specific temporal sequence as shown in Figure 4a. The bending displacement corresponding to each irradiation step is measured as the distance traveled by the cantilever tip (see Methods and Figure S4 for details). In the first 40 s, a UV-blue exposure actuates the bending motion (whose direction does not depend on the illuminated side), reaching its maximum amplitude (∼2 μm for 6-NO_2_–BIPS/PMMA and ∼16 μm for 6-NO_2_–BIPS/PMMA-rGO) within the first 5 s (Figure 4b,d). During illumination by UV-blue light (Figure 4b,c), the SP molecules embedded in PMMA are gradually converted to MC, gaining a distinct purple color with characteristic time, τ_UV_ = 26 ± 2 s, as measured by optical absorption (Figure S5).

**Figure 4:**
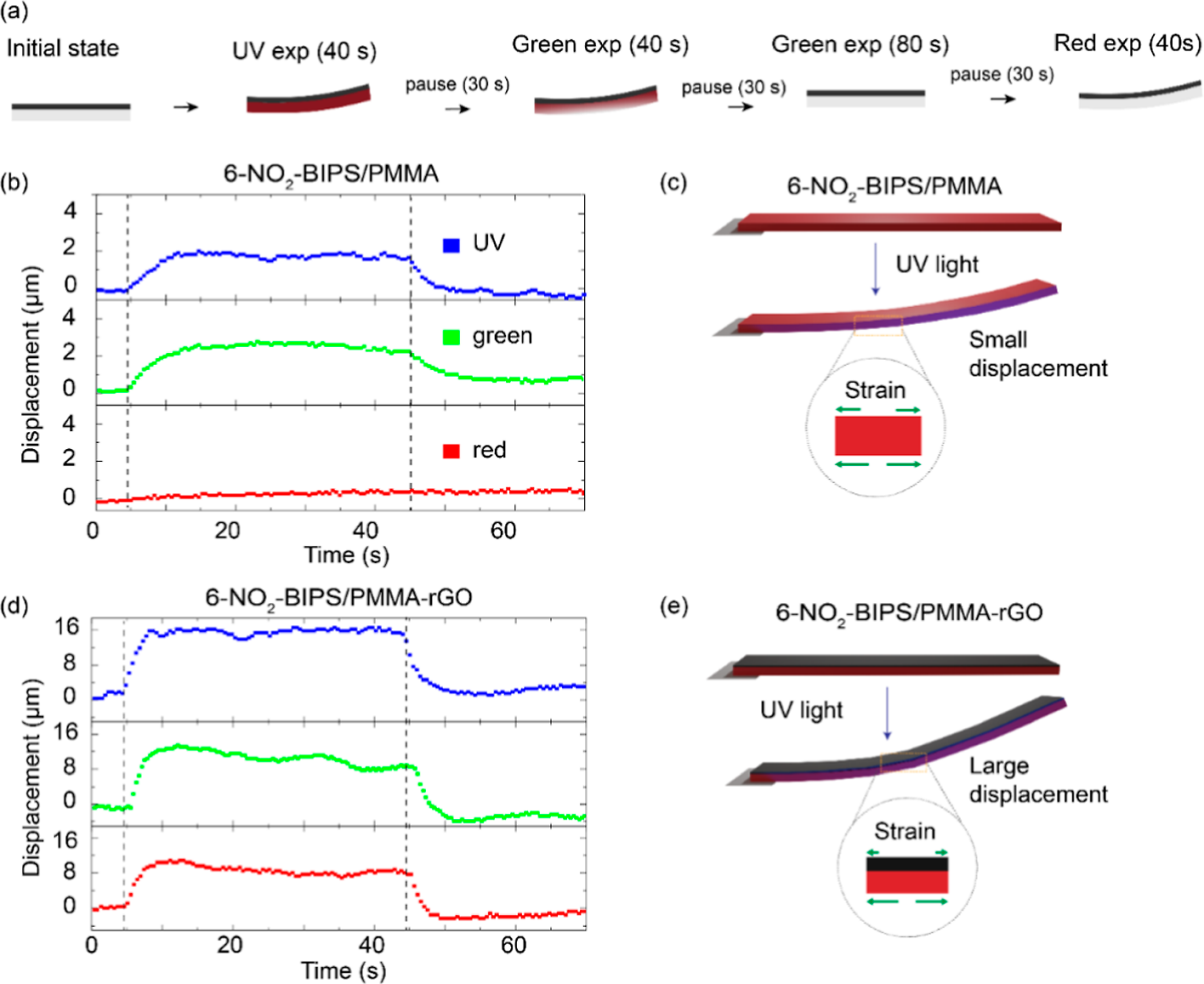
(a) Illumination temporal sequence. (b–e) Displacement induced by UV-blue (405 nm wavelength), green (561 nm wavelength), and red (638 nm wavelength) irradiation (3.3 mW cm^–2^), with the temporal sequence in (a), for 6-NO_2_–BIPS/PMMA (b,c) and 6-NO_2_–BIPS/PMMA-rGO cantilevers (d,e). Bending is largely independent of the illuminated side in the 6-NO_2_–BIPS/PMMA (c). UV light is sent onto the photochromic side of 6-NO_2_–BIPS/PMMA-rGO (e).

Afterward, the UV-blue illumination is switched off and the cantilever relaxes to its initial position within ∼5 s, keeping its purple color for a longer time. In fact, the lifetime of the MC→SP back-relaxation at room-temperature takes as long as tens of minutes (Figure S6). This slow optical reconfiguration clearly indicates that isomerization-induced geometrical changes are mostly uncoupled from the relaxational bending dynamics. Instead, the photoactuation of 6-NO_2_–BIPS/PMMA could be driven by nonuniform thermomechanical properties in the layered system. A mismatch of photoisomerization and bending mechanisms has been previously observed in heavily doped systems with low glass-transition temperature, and attributed to aggregate formation and suppression dynamics.^9^ By absorbance measurements (Figure S5a,b) and penetration depths for UV-blue light (Table S1), we estimate that 60–86% of photons are absorbed in the 60 μm-thick photochromic layer at the illuminated side of the cantilever. While a residual thermal gradient might be formed across the thickness of the cantilever upon illumination, due to decay of vibrationally hot molecular states and the small thermal conductivity of PMMA,^32^ nonuniform thermo-mechanical properties can be dominant in determining the direction and amplitude of the bending behavior (see Supporting Information for details). This important property will be better discussed below. Moving forward in the activation sequence, green illumination is then turned on for 40 s, which also leads to a displacement of the cantilever, as shown in the middle panel of Figure 4b. At this stage, MC molecules absorb green photons and are back-converted to the SP form, with analogous local heating mechanisms taking place. Additional exposure by green light for 80 s resets the cantilever to its initial state, removing any nonback-converted MC component and making the 6-NO_2_–BIPS/PMMA system not responsive to further irradiation with visible light (both green and red, bottom panel of Figure 4b).

In fact, the bending mechanism and the mechanical relaxation that are largely uncoupled from MC→SP back-relaxation can be advantageous for cycling, allowing in principle multiple activations to be primed by green light (absorbed by the retained, long-living MC component) after the initial UV activation. Quantitative data of the MC absorption coefficient of MC at 561 nm start from a maximum of 500 cm^–1^ immediately after the initial UV activation, decreasing with a maximum, initial rate of 16 cm^–1^/s under green irradiation (Figure S5b,d). Hence, for multiple bendings, a sequence of many green irradiations can be used until, at a given total time of green irradiation of ∼80 s, the MC component is substantially depleted, and one should come back to UV light to restore it. Properly designing the sequence of irradiation steps, with light at different wavelengths to match the molecular absorption spectrum, might allow one to keep cycling well-coupled with the time scale of the mechanical bending response. This relevant property is also supported by the stable absorption of the photochromic components upon cycling (Figure S7).

The addition of the top rGO layer is found to enhance the bending motion (by up to ∼8 times, favored by the mismatch in the coefficient of thermal expansion, CTE, i.e., ∼1.5 × 10^–5^ K^–1^ for rGO and ∼5 × 10^–5^ K^–1^ for PMMA, respectively), and to enable photoresponse also by red light (Figure 4d,e). Experiments illuminating samples without 6-NO_2_–BIPS (i.e., undoped PMMA and PMMA-rGO cantilevers) with the same optical sequence are also carried out (Figure 5). PMMA-rGO cantilevers are found to be activated with all used wavelengths, but with smaller amplitude (∼3 μm maximum displacements) and longer actuation times (∼8 s) compared to 6-NO_2_–BIPS/PMMA-rGO, highlighting the synergy of the two absorbing materials in enhancing actuation. This enhancement is also measured by red illumination, which could be explained by light scattering from aggregates that might support large rGO absorption. Exemplary cycles of bending of a 6-NO_2_–BIPS/PMMA-rGO cantilever during a sequence of alternating UV and green light irradiation steps are shown in Figure S8, showing good stability of the bending amplitude.

**Figure 5:**
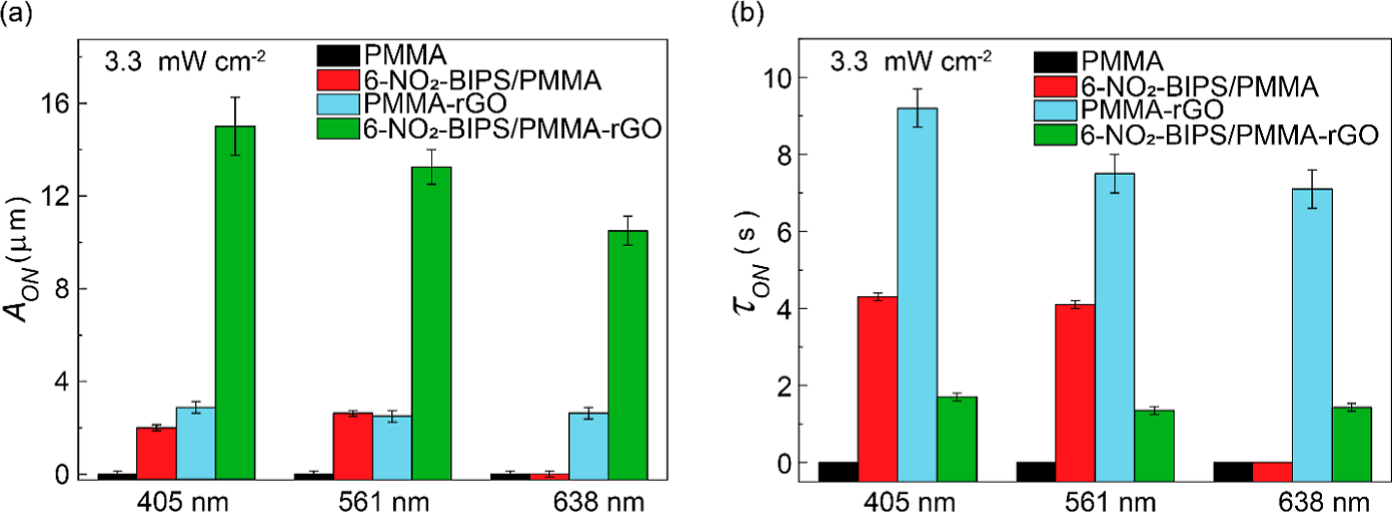
Bending parameters of four different systems irradiated with UV-blue (405 nm wavelength), green (561 nm wavelength), and red (638 nm wavelength) lasers (3.3 mW cm^–2^). (a) *A*_ON_: maximum amplitude of photoinduced displacement. (b) τ_ON_: characteristic actuation time.

To further corroborate the envisaged photothermal mechanisms of actuation, we acquire real-time thermal images of the cantilever surface (polymer side), upon switching on and off the various illumination sources. An exemplary temporal evolution of temperature for the 6-NO_2_–BIPS/PMMA-rGO photoactuator under UV-blue light illumination is shown in Figure 6. Varying the incident light intensity, a linear slope coefficient of (0.20 ± 0.01) K mW^–1^ cm^2^ is measured for 6-NO_2_–BIPS/PMMA-rGO heating (Figure S9a). The temperature increase measured upon switching light on, and the characteristic temperature rise times, are summarized in Figure S9b,c for all the fabricated devices and used wavelengths. Similar increases in temperature (*A*_Δ*T*_ ∼ 0.7 K) and thermal rise times (τ_Δ*T*_ = 2–4 s) are measured in the various samples (at the wavelengths at which they absorb radiation). The analogous heating-bending characteristic time scales, together with Finite Element Method (FEM) simulations (Figure S10 and Table S2), support photothermal effects as mechanism relevant for actuation. After switching off the UV-blue laser, the decrease of temperature occurs on a time scale of a few seconds (Figure 6), similarly to the mechanical relaxation to the original position (top panels in Figure 4b,d), while the photochromic dopants largely remain in their MC states (Figure S6).

**Figure 6:**
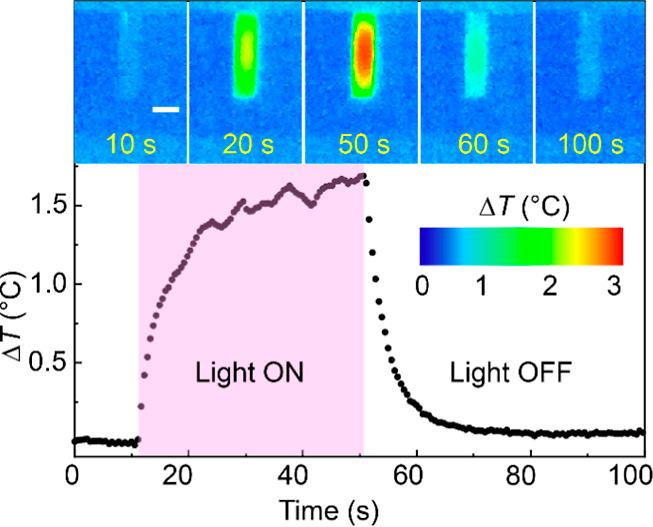
Temperature increase of a 6-NO_2_–BIPS/PMMA-rGO cantilever upon 40 s UV-blue illumination (405 nm, 7 mW cm^–2^, laser illumination), with respect to ambient temperature. The top insets show a sequence of infrared maps of the cantilever, measured at different times. Scale bar: 3 mm.

Using varied iron masses (∼25–95 mg), positioned on the cantilever tip (schematized in Figure 7a), the lift force exerted by these cantilevers can be estimated as a function of the incident power (Figure 7b, blue dots). For a given light intensity, the lift force is equal to the weight of the heaviest mass where the cantilever moves upward,^16^ detaching from the supporting glass (see Figure 7a). Finally, the tip displacement, without added masses, is measured in the same range of powers (Figure 7b, red dots). Both force and mass-free displacement show a linear response, reaching a maximum value of 1.9 ± 0.2 mN and 207 ± 3 μm at 92 mW cm^–2^, respectively. A summary of the found force and displacement values, together with those of other light-controlled, film-based, or bilayered actuators, is shown in Table S3. Classes of used materials are ample and include hydrogels with relatively slow dynamics (scale of minutes),^13,14^ other carbon-based systems (nanotubes, graphene oxide, stacked assemblies, etc.) layered with polycarbonates, polyethylene, or PMMA,^16,17,23^ and very soft systems, such as those based on or encompassing polydimethylsiloxane^18–20,22^ showing relatively large displacements. Overall, the performance of different architectures and geometries is difficult to compare, and lift forces are not always measured and reported. However, a combination of mN exerted forces, displacement in the range of hundreds of μm, and relatively fast (s-scale) actuation times, as those measured here, makes 6-NO_2_–BIPS/PMMA-rGO devices very good in the framework of photocontrolled polymeric microsystems. In terms of potential applications, this performance can be useful for many fields, including biomimetic motor devices and biomedical technologies.

**Figure 7:**
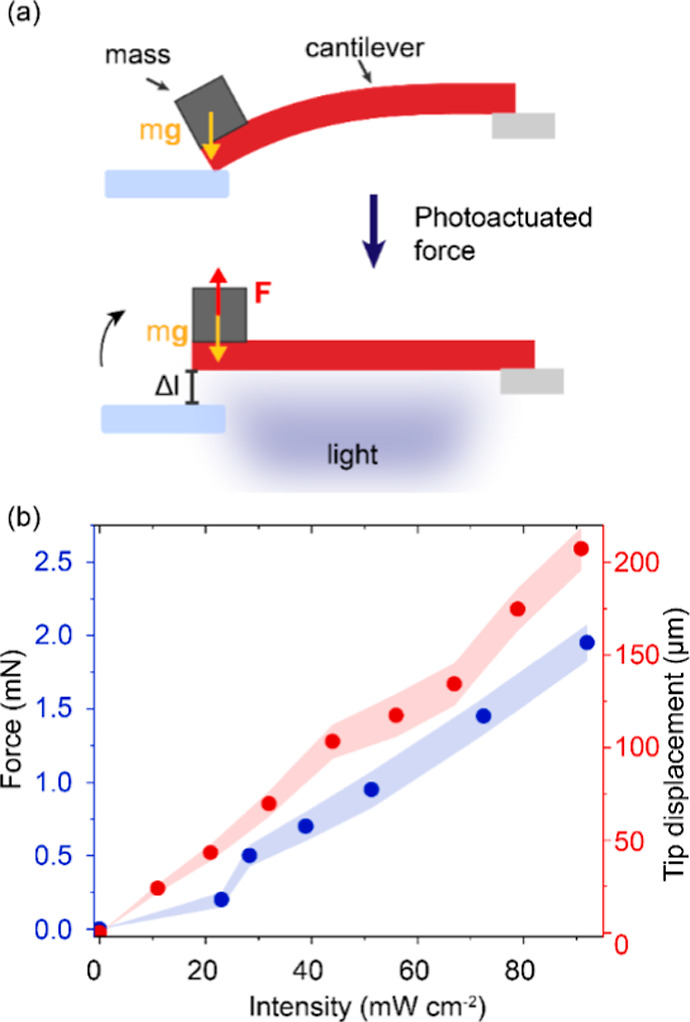
(a) Schematics of the setup to measure the lifting force capability. (b) Force (blue dots) and tip displacement (red dots) of the 6-NO_2_–BIPS/PMMA-rGO vs incident intensity (385 nm wavelength, up to 92 mW cm^–2^, LED illumination). The shadowed areas indicate experimental uncertainty from performed measurements. We emphasize that the UV source used for these measurements is different from that used for thermal imaging in Figure 6.

Overall, it is worthwhile to notice that even a very modest temperature rise in glassy photochromic systems can interact with an internal stress landscape set by processing history. Therefore, a very useful elucidation scheme for the fundamental mechanisms of light-triggered bending in photochromic-doped polymers would require considering the photochemical state of the photoswitch in the host matrix^5,6,8^ but also the thermomechanical sensitivity of films, as well as their processing imprints, which can introduce depth-dependent property gradients.^33–35^ For instance, previous studies highlighted that PMMA can show large variations of CTE depending on structural features and local free volume fraction,^36^ as well as retain residual solvent presence and display solvent-dependent relaxational behavior^37^ and thickness-dependent density variations^34^ upon casting from solution. To examine more in depth whether a through-thickness asymmetry in the 6-NO_2_–BIPS/PMMA system can account for the observed bending response, we implement a three-layer FEM model consisting of 5/50/5 μm top/core/bottom regions, where the outer layers represent effective near-surface regions and the central layer a bulk-like 6-NO_2_–BIPS/PMMA core (Figure S11). In this framework, the top and bottom layers are assigned weak and opposite-sign variations relative to the core, modulating their relevant thermo-mechanical properties, namely, either CTE or Young’s modulus (Table S4). The structure with CTE varying by ± 7.5% with respect to the core is found to lead to deflection values (∼2 μm deflection under 3.3 mW cm^–2^ UV illumination) in excellent agreement with experimental results (top panel in Figure 4b). Instead, variations in Young’s modulus are not found to significantly affect the bending (Table S4). These results support depth-dependent property gradients as an effective thermal-strain trigger that governs the actuation in the polymeric systems.

The hybrid strategy of combining SP-doped polymers with graphenic layers like rGO can offer a distinct advantage: the active molecular component provides wavelength-dependent absorption and optical state control, while the rGO supplies broadband photothermal triggering and interfacial contrast. The photothermal conversion efficiency of our system, defined as the ratio of the generated thermal power to the input optical power^28^ and estimated by analyzing the postillumination thermal relaxation (Figure S12), has a lower-limit value of about 55% (see Section S12 in the Supporting Information for details).

Importantly, the response of relatively thick polymer cantilevers cannot be inferred directly from ultrathin-film intuition because processing and interfaces can imprint depth-dependent structure and properties, including density, modulus, and thermal expansivity, so that a nominally uniform film can behave as an intrinsic bimorph. These properties have an impact on the bending directionality, amplitude, and response time, and their rationalization can thus allow for a more accurate design of remotely and precisely controlled soft-actuator components.

## Conclusion

In summary, light-triggered 6-NO_2_–BIPS/PMMA-rGO is proposed as an easy-to-fabricate and effective bilayer architecture for soft actuators. While presenting unique photothermal properties that cannot be anticipated by thin-film behavior and very weak internal temperature gradients, these hybrid systems combine high interlayer thermomechanical contrast with intrinsic mechanical amplification of photoinduced strain mismatch. These synergistic mechanisms lead to significantly enhanced displacement compared with purely polymeric and purely photochromic cantilevers. On one side, this study adds new knowledge about photochromic-doped polymer systems, suggesting the existence of actuation pathways associated with built-in asymmetries along the thickness direction. On the other side, the rGO layer is found to increase the overall practicability of the entire system. By virtue of rapid heat spreading, broadband photothermal triggering, which leads to a remarkable multi-photoaddressable behavior, faster thermal rise time, and faster actuation times, rGO might play an important role in determining the ultimate bending performance. Applications of these results can be relevant for various photocontrolled components, as well as for biomimetic and biomedical devices with precise structure–function design of optical triggering and propelling schemes.

## Methods

### Fabrication of 6-NO_2_–BIPS/PMMA-rGO Bilayer Actuators

The photochromic layer is realized by drop casting a chloroform solution of 6-NO_2_–BIPS and PMMA onto a glass substrate (18 × 18 mm^2^). To this aim, 200 mg of PMMA (Sigma-Aldrich, Mw: 120 k, glass transition temperature: 105 °C) and 5 mg of 6-NO_2_–BIPS (Sigma-Aldrich) are dissolved in 1 mL of chloroform. The used 6-NO_2_–BIPS concentration is chosen as a value that, while being high enough to allow for intense photoabsorption and consequent bending, leads to absorption of the incident light for depths above a few tens of μm, for all the used wavelengths (see Table S1 in the Supporting Information). This property is relevant to favor the exploitation of the full 6-NO_2_–BIPS-doped volume as well as the penetration of the incident light as closely as possible to the rGO layer, i.e., to rule out tight confinement of the excitation near the illuminated surface. The resulting solution is vigorously stirred, and a volume of 110 μL is then deposited on glass. To obtain a homogeneous film, the coated substrate is placed overnight in a Petri dish while in contact with chloroform vapors. The resulting film has a thickness of 60 μm. The rGO layer is obtained by drop casting a dispersion of rGO in ethanol (0.5 mg mL^–1^) onto the 6-NO_2_–BIPS/PMMA film. Numerous samples are realized using different volumes of the rGO dispersion, drop-casted on the sample areas. Samples are then heated at 50 °C on a hot plate to enhance solvent evaporation. Finally, the bilayer film is gently lifted from the glass substrate and cut into 10 × 2.5 mm^2^ cantilevers by a scalpel.

### Morphology, Optical Properties, Force, and Deflection Characterization of Actuators

Optical microscopy (Olympus BX52), confocal microscopy (Olympus FV1000, 20× objective, numerical aperture 0.75, excitation wavelengths 543 and 458 nm), and SEM (FEG-SEM, Merlin from Zeiss) are employed for characterizing the properties of the photocontrollable cantilevers. A spectrophotometer (mod. Lambda950, PerkinElmer) is used to measure optical absorption. The rGO surface coverage is analyzed using a threshold filter on optical micrographs: if the gray scale value of a pixel is below the threshold, the value of the pixel is set to 0 (black), otherwise the pixel is set to 255 (white). The number of black pixels is then divided by the number of total pixels to estimate the fractional area covered by rGO. Raman spectra are collected with a Renishaw InVia confocal microRaman system, using a 785 nm laser, focused on the sample through a 50× objective. Two setups are used to measure cantilever bending. The first setup (Figure S4) is employed to measure the small amplitude bending (few tens of μm) in 6-NO_2_–BIPS/PMMA, either with or without top rGO. A multicolor laser source (mod. L6Cc combiner, Oxxius) is used to illuminate the cantilevers with three separate wavelengths (405, 561, and 638 nm), each one coupled to a single mode optical fiber. After passing through a system of lenses and mirrors, the output beam is directed perpendicular to the cantilever surface. The lens system is designed to shape the beam in an elliptical spot (spot size 1.05 × 0.35 cm^2^), such that an almost uniform irradiation of the sample is achieved. A laser emitting at 785 nm is used to assess the bending motion, since both SP and MC isomers do not show absorption at such wavelength. The laser beam is sent onto the cantilever surface at about 45°, and the intensity of the reflected component is measured by a 4-quadrants photodiode (QP50–6SD2-DIAG, First Sensor) and an oscilloscope. The variation of the signal, associated with the difference of the intensity detected by the upper-half and that detected by the lower-half of the photodetector, is linearly correlated to the displacement of the cantilever tip. Drift signals corresponding to up to ∼0.7 μm (2.0 μm) bending are estimated for this setup for 6-NO_2_–BIPS/PMMA (6-NO_2_–BIPS/PMMA-rGO) samples, which can be associated with residual stress, moisture absorption, and long-term drift in the optoelectronic detection, as reported for actuators and atomic force microscopy equipment with similar optical detection methods,^38,39^ as well as to uneven reflection on the cantilever body. The second setup is employed for conducting force measurements. In this configuration, the cantilever is positioned horizontally, with the light generated by an UV LED (LZ1–00UB00 LED-Engin Osram, 385 nm, maximum power output 1200 mW with 68° viewing angle) impinging from the bottom of the device at 1 cm distance. The displacement of the cantilever tip is quantified through images captured by a CMOS camera (DCC1645C Thorlabs). For the force measurements, a series of iron masses (25–95 mg) are placed on the free tip of the cantilever, which rests on a glass substrate. Under a specific light intensity leading the cantilever at the threshold for upward movement, its lift force equals the gravitational force exerted by the mass (Figure 7a).

### Thermal Imaging

The temperature and the thermal images of photoactuators during exposure to light are acquired by using an infrared camera (A655sc, FLIR).

### Simulations

FEM simulations are performed with Comsol Multiphysics v6.1. Three physics modules are combined in a coupled time-dependent or stationary solver, namely, “Heat Transfer”, “Solid Mechanics”, and “Radiative Beam”. A null displacement is imposed at the anchored edge of the cantilever, and an effective heat-transfer coefficient, 12 W m^–2^ K^–1^, is applied to the whole exposed heat-exchange area (see Section S12). Heat fluxes at the cantilever top and bottom surfaces are set by the air convective heat transfer coefficient. Illumination sources are simulated with a Gaussian beam profile, with the appropriate beam width and considering collimated light for the sake of simplicity. The top surface is meshed with square face elements, swept through the cantilever thickness to complete the 3D mesh. The material properties used for the simulations are listed in Table S2.

## Supplementary Material



## Notes

Additional details and calculations on fabrication of the 6-NO_2_–BIPS/PMMA-rGO bilayer cantilever, optical transmission properties, SEM and laser confocal microscopy measurements, absorbance measurements, thermal analysis, and simulations (PDF)

## §

Sustainable and Bioinspired Materials Max Planck Institute of Colloids and Interfaces Potsdam D-14476, Germany

## ∥

Institute of Nanotechnology, CNR-Nanotec Institute of Nanotechnology, National Research Council and Tecnomed Puglia – Technopole for Precision Medicine (Biotech Lecce Hub) Lecce I-73100, Italy.
