# Supplementary material for: Light-Triggered Bending in Photochromic/Graphene Oxide Bilayers via Synergistic Photo-Thermal Actuation and Mechanical Amplification
Source: ACS Appl Mater Interfaces. 2026 Jun 3;18(23):33171–8. doi: 10.1021/acsami.6c04058 (PMC13288387; doi:10.1021/acsami.6c04058)
Supplement: Supplementary file 1 [file am6c04058_si_001.pdf]

Supporting Information

Light-Triggered Bending in Photochromic/Graphene Oxide Bilayers via Synergistic Photo-Thermal Actuation and Mechanical Amplification

Lorenzo Lavista[†,‡], Leonardo Vicarelli[†,], Maria Murace[†,‡,+], Alberto Portone[‡,++], Filippo Fabbri[‡], Federica Bianco[‡], Stefano Roddaro[†,‡], Alessandro Tredicucci[†,‡], Andrea Camposeo[‡], Luana Persano[‡], Dario Pisignano[†,‡,*]*

[†] Dipartimento di Fisica, Università di Pisa, Largo B. Pontecorvo 3, I-56127 Pisa, Italy.

[‡] Istituto Nanoscienze-CNR, NEST-SNS, Piazza S. Silvestro 12, I-56127 Pisa, Italy

[+] present address:
Sustainable and Bio-inspired Materials
Max Planck Institute of Colloids and Interfaces
Potsdam D-14476, Germany

[++] present address:
Institute of Nanotechnology CNR-Nanotec Institute of Nanotechnology, National Research Council and Tecnomed Puglia – Technopole for Precision Medicine (Biotech Lecce Hub)
Lecce I-73100, Italy

*Corresponding authors, leonardo.vicarelli@unipi.it, dario.pisignano@unipi.it

Keywords: photochromic molecules, reduced graphene oxide, optical control, soft actuators, bilayer devices

Section S1 - Fabrication of the 6-NO_2-BIPS/PMMA-rGO bilayer cantilever

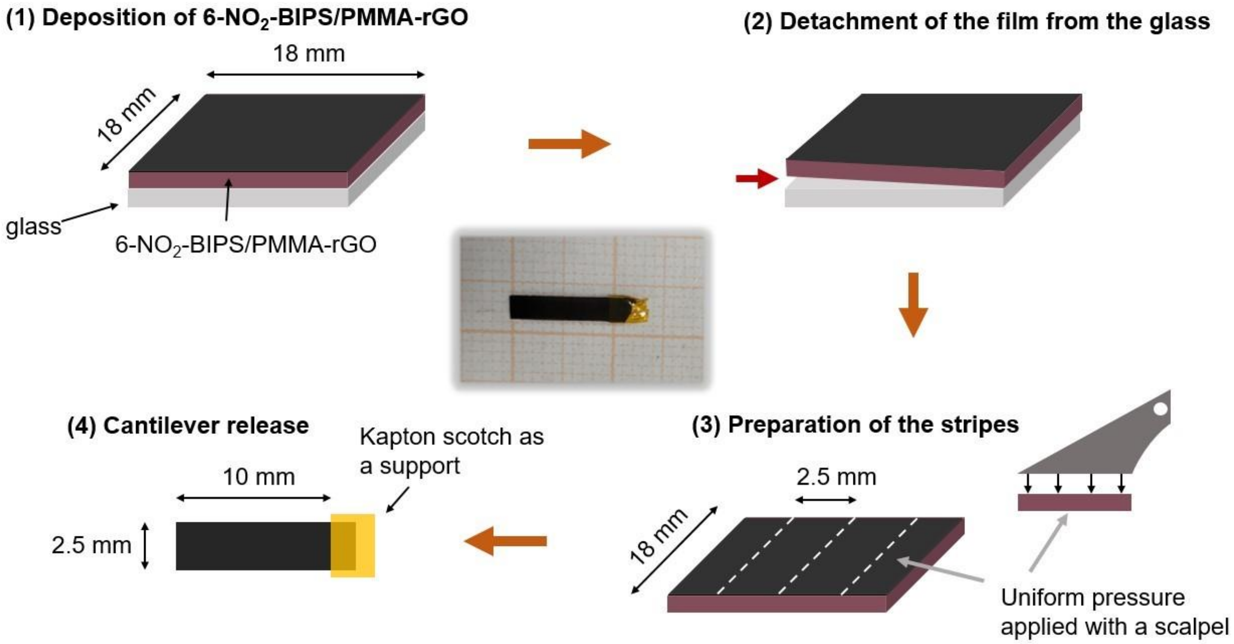

Figure S1. Fabrication process of the bilayer cantilever. (1) 6-NO_2-BIPS/PMMA and rGO films are deposited on a glass substrate. (2) The films are gently detached from glass. (3) The 6-NO_2-BIPS/PMMA-rGO films are cut in form of stripes. (4) The stripes are positioned near the edge of Kapton scotch. A photograph of a cantilever is shown in the center.

Section S2 - Optical transmittance of 6-NO_2-BIPS/PMMA-rGO bilayer

The optical transmission properties of the samples are investigated by means of a spectrophotometer (mod. Lambda950, Perkin Elmer). The transmittance, T_R, is calculated as the ratio between the intensity of light transmitted by the sample (I_t) and the incident one (I_0), $T_R = I_t / I_0$. The spectrometer beam was shined on the PMMA side of the bilayer.

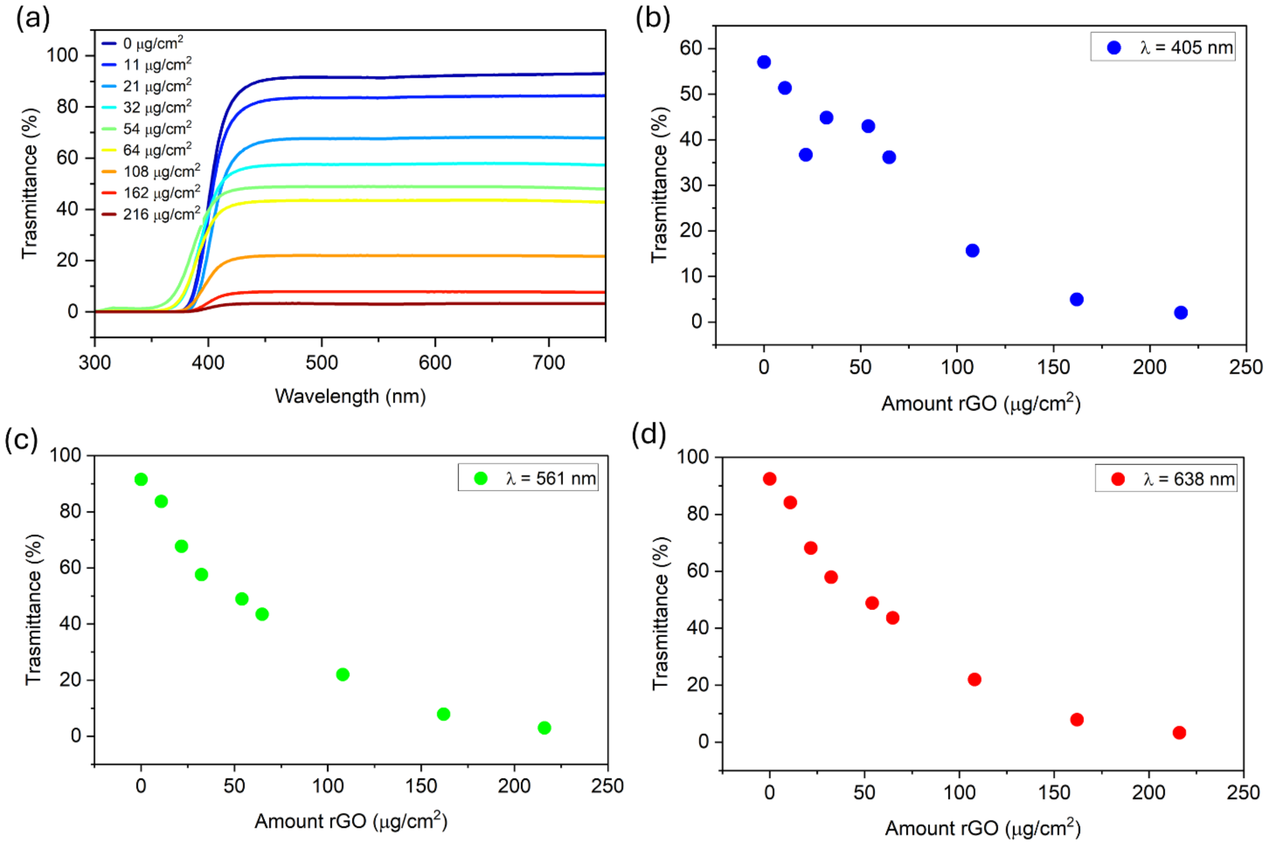

Figure S2. (a) Optical transmittance of samples realized by depositing different amounts of rGO dispersion (measured as μg cm^{-2}) on 6-NO_2-BIPS/PMMA. Samples are measured within 30 minutes after fabrication, and green light from a torch (5 mW/cm^2, 120 s) is used to illuminate them and remove residual pristine MC components prior to spectrophotometry experiments. (b)-(d) Dependence of the optical transmittance on the amount of rGO at wavelength of the incident light of (b) 405 nm (c) 561 nm and (d) 638 nm, respectively. A multicolor laser source is used to analyze the optical transmission at given wavelengths (b-d).

Section S3 – SEM and laser confocal microscopy of the 6-NO_2-BIPS/PMMA-rGO bilayer

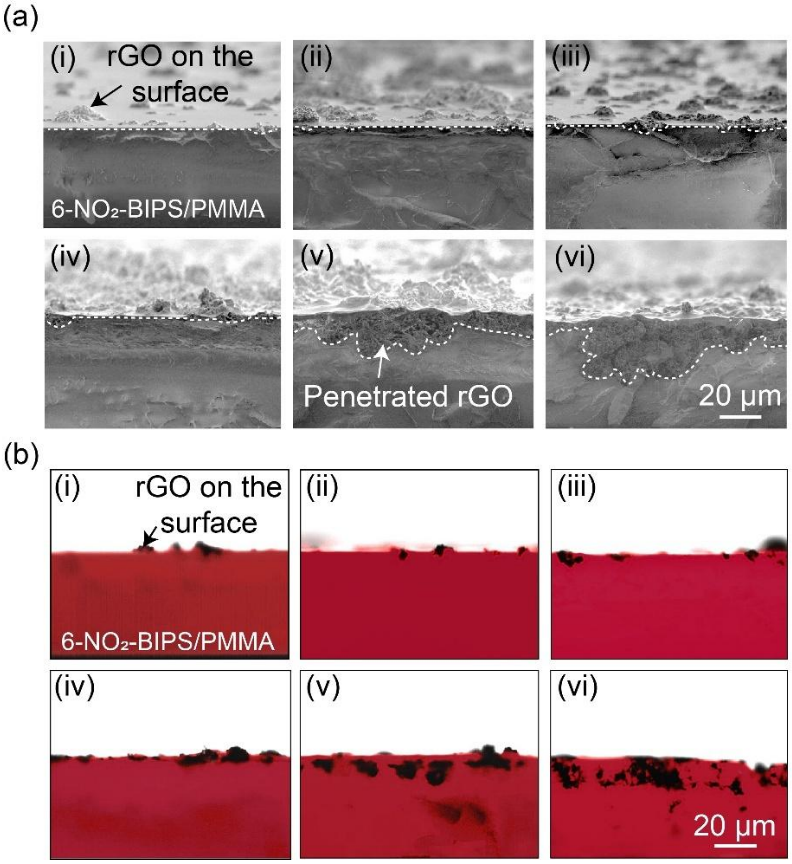

Figure S3. (a) SEM micrographs and (b) confocal microscopy cross-sectional images of the 6-NO_2-BIPS/PMMA-rGO bilayers for various amounts of cast rGO: (i) 11 µg cm^{-2}, (ii) 32 µg cm^{-2}, (iii) 64 µg cm^{-2}, (iv) 108 µg cm^{-2}, (v) 162 µg cm^{-2}, (vi) 216 µg cm^{-2}. In (b), rGO particles are represented by the black regions while the photochromic-doped layer corresponds to the red regions.

Section S4 - Optical set-up for small-deflection measurements

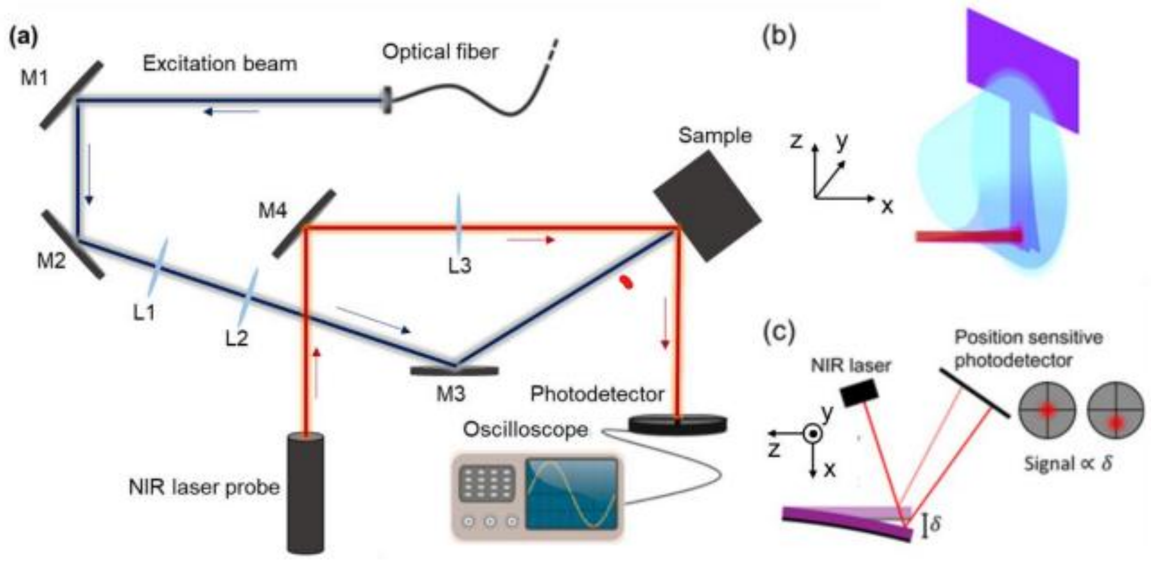

Figure S4. (a) Scheme of the set-up for the detection of small bending of the cantilever. M1, M2, M3 and M4: mirrors. L1, L2, and L3: lenses. (b) Illustration of the bending mechanism of the cantilever. (c) Schematic illustration of the four quadrant-photodiode and of the bending measurements.

Section S5 - Absorbance measurements of 6-NO_2-BIPS/PMMA film

We perform optical absorbance measurements upon using different UV-blue or green light exposure times, to determine the conditions for a full SP→MC and MC→SP conversion. Here, the coloring characteristic time (τ_{UV}) is considered, as the time in which the SP dopant undertakes full conversion to the open form, MC upon UV-blue illumination (405 nm). Similarly, τ_{green} indicates the characteristic time of the reverse process, namely the characteristic time needed for MC to revert to SP upon illumination with green light (561 nm). To determine τ_{UV} and τ_{green}, absorbance spectra are collected on a 60 µm thick 6-NO_2-BIPS/PMMA film, right after sample illumination for different time intervals with UV or green laser light (3.3 mW cm^{-2}). The results of these measurements are reported in Figure S5a,b. The MC absorbance peak at (570±1) nm clearly emerges upon UV illumination. Calculating the integrated area (I_A) under the MC absorbance peak, characteristic transition times τ_{UV} and τ_{green} are estimated by fitting I_A as a function of the exposure time. The following equations can be used to obtain the characteristic transition times, with t indicating the initial instant of irradiation at a given wavelength:

$$I_{A,UV}(t) = I_{0,UV} + A_{UV}\left(1 - e^{-t/\tau_{UV}}\right)$$

$$I_{A,green}(t) = I_{0,green} + A_{green}\, e^{-t/\tau_{green}}$$

where the subscripts indicate the coloring (SP→MC) and the reverse (MC→SP) processes, $I_{0,UV/green}$ are the absorbance offsets, while $A_{UV/green}$ contains the information about the maximum absorbance in the MC state. Experimental data and the resulting fitting curves are reported in Figure S5c,d. The characteristic times obtained from the exponential fits are τ_{UV} = (26±2) s and τ_{green}= (30±1) s. From these measurements, penetration depths at 405 nm, 561 nm and 638 nm wavelengths (corresponding to the three used laser sources) are also extracted for the SP (fully transparent in the visible) and MC (fully colored) states. Results are reported in Table S1.

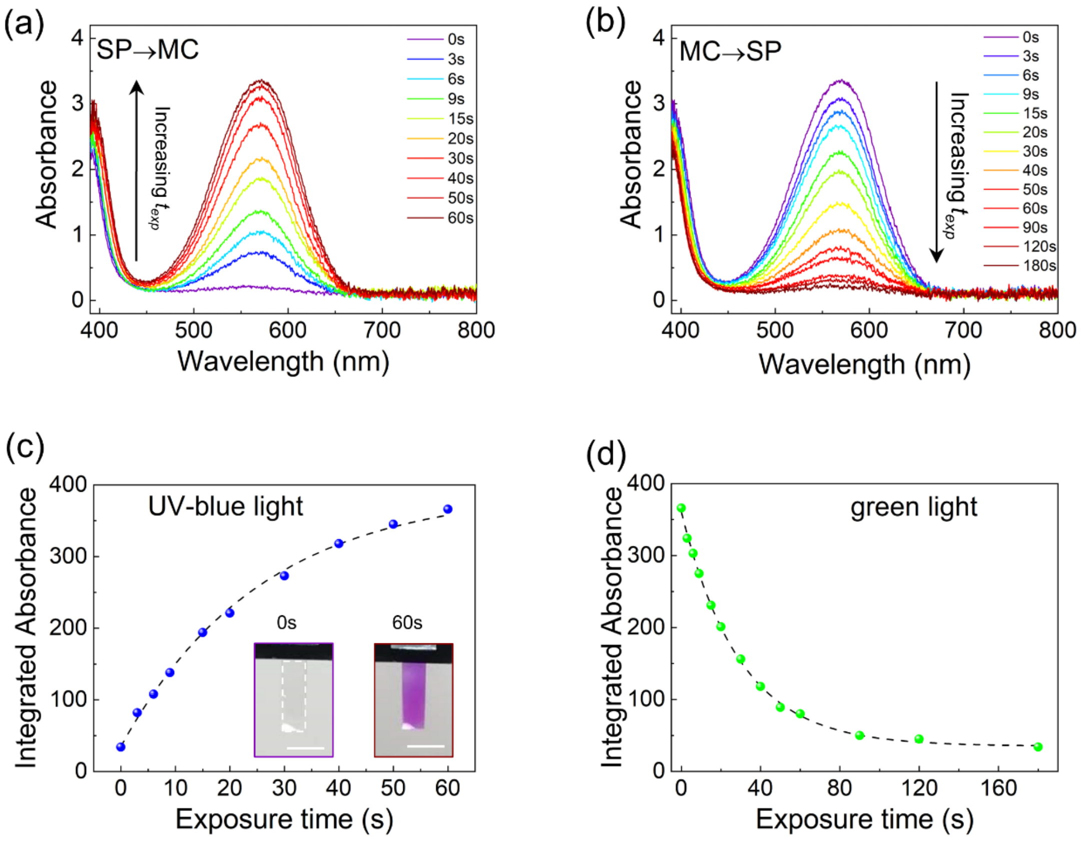

Figure S5. (a-b) Absorbance spectra of 6-NO_2-BIPS/PMMA at different exposure times for (a) UV-blue laser illumination and (b) green laser illumination (both with 3.3 mW cm^{-2} intensity). The arrows indicate the increase of the exposure time (t_{exp}). (c-d) Integrated absorption *vs*. exposure time for (c) UV-blue and (d) green exposure, respectively. Insets in (c): images of the cantilever before (transparent) and after 60 s of UV-blue illumination (purple color). Scale bar: 5 mm.

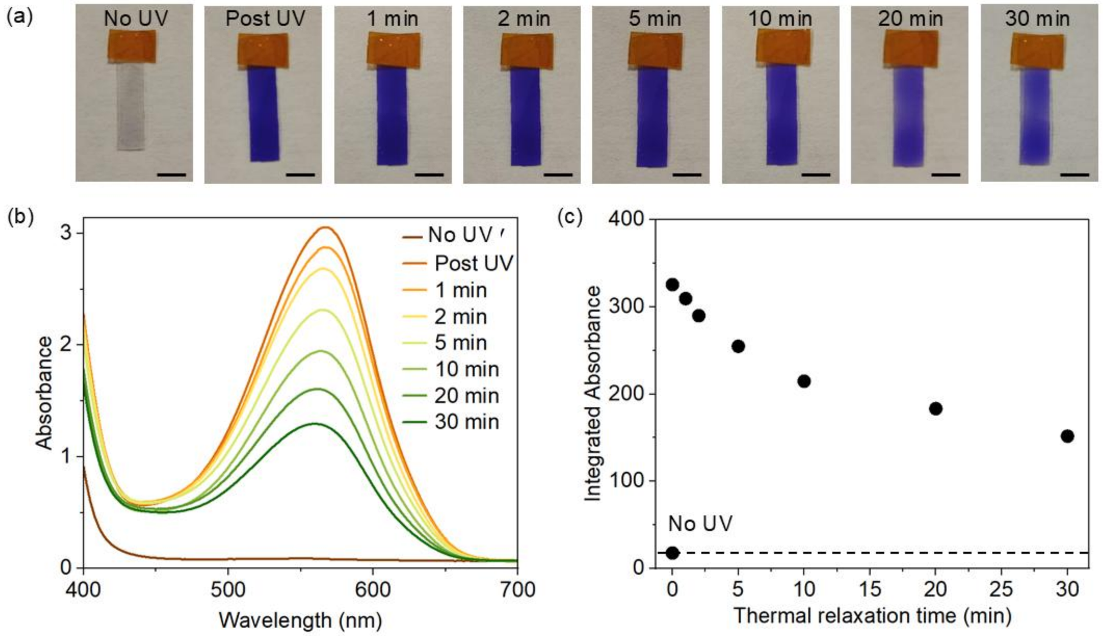

Figure S6. Photochromic recovery after UV irradiation (wavelength: 405 nm, intensity: 3.5 mW cm^{-2}). (a) Photographs of a 6-NO_2-BIPS/PMMA film before UV exposure, immediately after UV irradiation, and after different relaxation times (1, 2, 5, 10, 20, and 30 min) upon switching off the UV light, showing persistent purple coloration associated with the MC state at room temperature. Scale bar: 3 mm. (b) Corresponding absorption spectra collected at varied relaxation times. (c) Integrated absorbance of the MC band *vs*. thermal relaxation time after UV switch-off. The dashed line indicates the baseline absorbance of the pristine sample.

Wavelength	Penetration depth	
	SP state (0 s of UV-blue illumination, see insets of Fig. S5c)	MC state (60 s of UV-blue illumination, see insets of Fig. S5c)
405 nm	65 µm	30 µm
561 nm	300 µm	20 µm
638 nm	770 µm	70 µm

Table S1. Penetration depth extracted from absorbance measurements.

Section S6. Cycling behavior

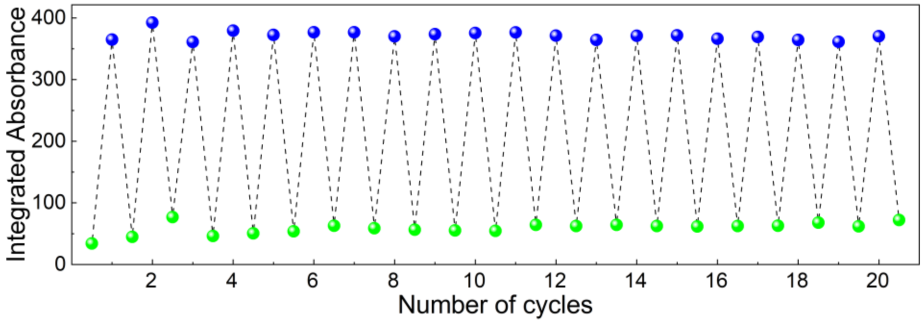

Figure S7. Integrated absorbance of the MC band for alternating steps of UV-blue laser (blue dots; wavelength: 405 nm, intensity: 3.3 mW cm^{-2} intensity) and green laser irradiation (green dots; wavelength: 561 nm, intensity: 3.3 mW cm^{-2} intensity) irradiation, showing good stability. The dashed lines are guides for the eye.

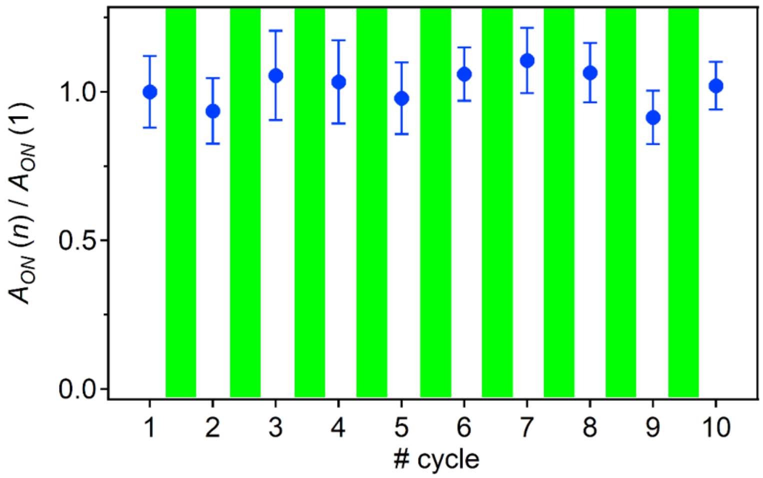

Figure S8. Cyclic variation of the maximum bending amplitude of displacement (A_{ON}) for 6-NO_2-BIPS/PMMA-rGO upon irradiation with UV-blue laser (wavelength: 405 nm, intensity: 3.3 mW cm^{-2}) for 40 s. The green shadowed areas highlight the irradiation to green laser (wavelength: 561 nm, intensity: 3.3 mW cm^{-2}) performed between two consecutive UV-blue laser irradiations to induce MC→SP convertion. Data are normalized to the maximum bending amplitude of displacement measured for the first cycle, $A_{ON}(1)$.

Section S7 - Control experiments and dependence of actuation on wavelength

Characteristic actuation times τ_{ON} and maximum displacement amplitudes A_{ON} are estimated by fitting the displacement as a function of the exposure time using the equation:

$$A(t) = A_{ON}\left(1 - e^{-t/\tau_{ON}}\right)$$

The fit is repeated for all four possible configurations of the cantilever composition (with/without rGO and 6-NO_2-BIPS), and for the three different wavelengths of the illumination source (UV-blue, green, red). Results are reported in Figure 5.

Section S8 - Thermal imaging and temperature dependence on wavelength

The characteristic temperature rise time, $\tau_{\Delta T}$, and maximum temperature amplitude, $A_{\Delta T}$, are estimated by fitting the temperature increase (averaged on the cantilever area, with respect to ambient temperature) as a function of the exposure time using the equation:

$$\Delta T(t) = A_{\Delta T}\left(1 - e^{-t/\tau_{\Delta T}}\right)$$

The fit is repeated for all the fabricated devices and used wavelengths. Results of thermal imaging and analysis are reported in Figures 6 and S9, respectively.

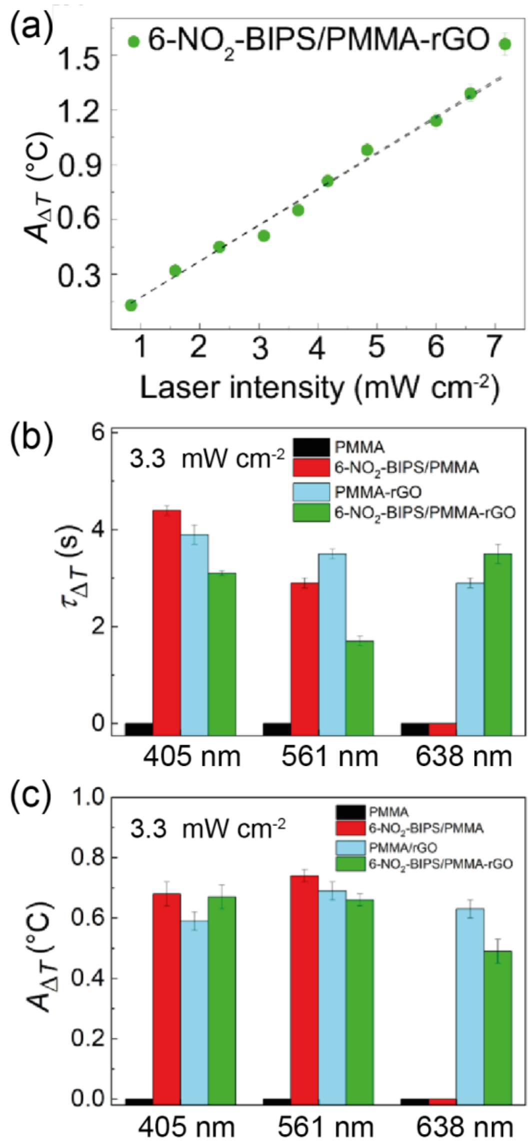

Figure S9. (a) $A_{\Delta T}$ *vs*. incident UV-blue (wavelength: 405 nm) laser intensity for 6-NO_2-BIPS/PMMA-rGO. The dashed line is a linear fit to the data. (b)-(c) Characteristic temperature rise times $\tau_{\Delta T}$ and maximum temperature amplitudes $A_{\Delta T}$, respectively, for all the fabricated configurations, irradiated with UV-blue (405 nm), green (561 nm) and red (638 nm) light (3.3 mW cm^{-2}).

Section S9 – FEM simulations of the bilayer cantilevers

Finite Element Method (FEM) simulations allow the expected displacement and temperature increase to be estimated upon assuming a purely photothermal actuation. Thermal losses are described by a single effective heat-transfer coefficient, 12 W m^{-2} K^{-1}, and considering a 55% photothermal conversion (see Section S12). In both simulations, with and without rGO, actuation times τ_{ONsim} are found to coincide with their respective thermal rise times $\tau_{\Delta Tsim}$.

For 6-NO_2-BIPS/PMMA irradiated with 3.3 mW cm^{-2}, a maximum temperature increase (averaged on the cantilever area) of $A_{\Delta Tsim}$ ~0.7 K and thermal rise time of $\tau_{\Delta Tsim}$ ~6 s are found from simulations, well in line with experimental values. A time-independent UV-blue absorption (e.g., with complete photoconversion of 6-NO_2-BIPS in the MC state), only leads to a slightly lower $\tau_{\Delta Tsim}$ value (~5 s). A gradient as low as 1 mK is found across the cantilever thickness, resulting in a 0.1 μm vertical tip displacement, which is considerably smaller than the measured values. This suggests that for 6-NO_2-BIPS actuators the photothermal pathways should be complex, namely thermal gradient across the cantilever thickness is not sufficient per se as driving mechanism, but it should instead be accompanied by non-uniform sample properties across the thickness. These non-uniform properties can be due to the fabrication process, and include, for instance, variable formation or suppression of aggregates, different chain mobility, and thermo-mechanical properties associated with local microporosity and density.

For the 6-NO_2-BIPS/PMMA-rGO device irradiated with 3.3 mW cm^{-2}, simulations show a similar $A_{\Delta Tsim}$ ~0.7 K and gradient of 1.7 mK across the cantilever thickness, but a faster thermal rise time $\tau_{\Delta Tsim}$~4.5 s, thanks to the additional thermal conductivity of rGO (Table S2). Simulated maximum tip displacement now reaches ~29 μm. With an irradiation intensity of 7 mW cm^{-2}, the maximum tip displacement reaches ~61 μm.

Exemplary heating and bending results under different values of the irradiation intensity (3.3 and 7 mW cm^{-2}) are shown in Figure S10.

Material parameter	PMMA value	Ref.	rGO value	Ref.
Thermal expansion coeff. (CTE)	5×10^{-5} K^{-1}	S1	1.5×10^{-5} K^{-1}	S2
Thermal conductivity	0.19 W m^{-1} K^{-1}	S3	10 W m^{-1} K^{-1}	S4
Heat capacity	1370 J kg^{-1} K^{-1}	S3	750 kg m^{-3}	S5
Density	1190 kg m^{-3}	S3	1000 kg m^{-3}	S5
Young Mod.	3 GPa	S5,S6	110 GPa	S5
Poisson ratio	0.35	S3	0.186	S5
Absorption coefficient (405 nm)	170 cm^{-1} @ 0 s of UV-blue illumination, see insets of Fig. S5c<br>345 cm^{-1} @ 60 s of UV-blue illumination, see insets of Fig. S5c	Table S1	920 cm^{-1}	Fig. S2b

Table S2. PMMA and rGO material parameters used in FEM simulations.

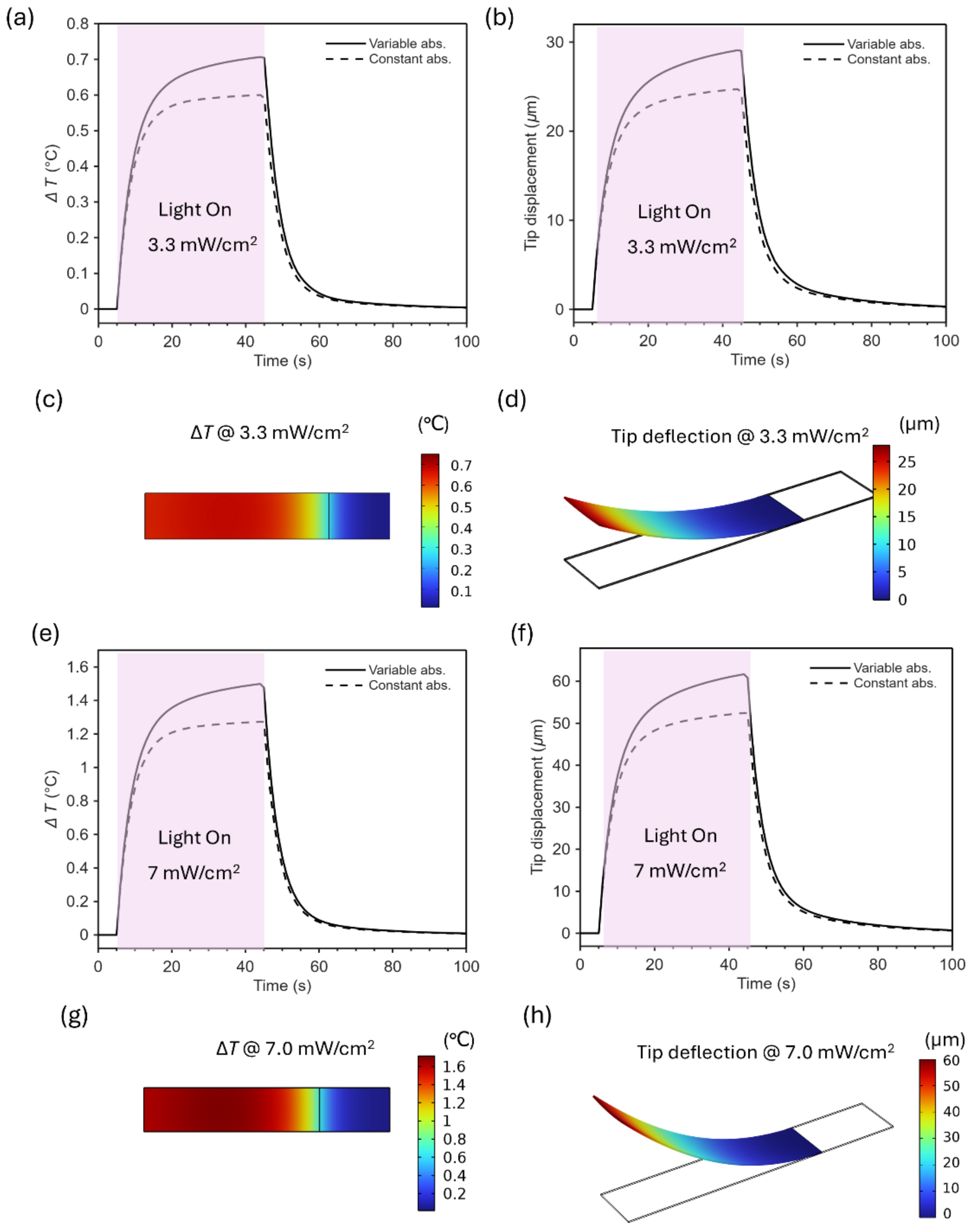

Figure S10. FEM simulation for the 6-NO_2-BIPS/PMMA-rGO cantilever. Simulated temperature increase (a,e) upon 40 s illumination with 3.3 mW cm^{-2} (a) and with 7 mW cm^{-2} (e), with and without variable absorption due to the gradual SP to MC conversion (cfr. Figure 4d, main text), and corresponding

simulated displacement (b,f). (c,d,g,h): Simulated maps of temperature increase and deflection (visually amplified × 100).

Section S10 – Comparison with literature on film-based and bilayered systems

Ref.	Material	Actuation mechanism	Light source	Geometry	Displacement / bending	Resp. time	Force
S5	PMMA/ Au Nanorods+GO composite bilayer;	Photothermal bilayer bending	405,532, 635, and 808 nm lasers, 50-80 mW cm^{-2}	cantilever 20×2×0.025 mm	~250 m^{-1} curvature	~0.1 s	~60 mN
S7	SP-doped photochromic polymer (PEMMA)	Photochemical/ photochromic dimensional change leading to bending	308 nm and 532 nm laser pulses up to 70 mJ cm^{-2}	cantilever 2.5×1.5× 0.06-0.12 mm	~500 µm tip displacement	~ 4 s	N/A
S8	Polycarbonate /Single-wall carbon nanotubes bilayer	Photothermal bilayer bending	white light, 100 mW cm^{-2}	disk, 25 mm diameter, 10 µm thickness	~90° bending	~0.5 s	N/A
S9	PE/stacked graphene bilayer	Photothermal bilayer bending	IR lamp 120 mW cm^{-2}	pre-rolled cantilever 5×1-5×0.01 mm	~1-2 mm^{-1} curvature unrolling	~0.4 s	lifts 10 times its own weight
S10	PDMS/graphene nanoparticles composite bilayer	Photothermal bilayer bending	808 nm laser, 2950 mW cm-2	cantilever 10×1×0.13 mm	~1400 µm tip displacement	~ 3 s	N/A

S11	PDMS/PP-PEDOT bilayer	Photothermal bilayer bending	808 nm laser, 198 mW total (~400 mW cm^{-2})	cantilever 10×5×0.07 mm	~150° bending (~20 mm tip displacement)	~2-5 s	N/A
S12	PDMS/RGO bilayer	Photothermal bilayer bending	365 nm, 20 mW cm^{-2}; 473 nm, 20 mW cm^{-2}; 808 nm, 1100 mW cm^{-2}	cantilever 10×2×0.04 mm	88° (UV), 73° (VIS), 156° (NIR) bending	~ 1 s	N/A
S13	PDMS/ PDMS+graphene composite bilayer	Photothermal bilayer bending	980 nm laser; 2000 mW cm^{-2}	cantilever 20×3×0.12 mm	~ 8 mm tip displacement	~1-2 s	N/A
S14	PNIPAM-SP1 / PNIPAM-SP2 bilayer hydrogels	Synergistic photoexpansion + photocontraction in bilayer hydrogel	450 nm LED, 78 mW cm^{-2}	cantilever 10×2×0.6 mm	~80° bending	6-10 min	Bending moment ~2.0×10^{-7} N·m (equivalent to 20 μN)
S15	SP-PAAm / PAAm bilayer hydrogels	Photo-swelling mismatch / self-folding bilayer hydrogel	470 nm LED, 50 W total power	cantilever 30×5×5 mm	~75° folding	15–30 min	N/A
This work	6-NO_2-BIPS/PMMA –rGO bilayer	Internal gradients and photothermal bilayer bending	405, 561, 638 nm lasers<br>385 nm LED 92 mW cm^{-2}	cantilever 10×2.5×0.06 mm	207 ± 3 μm tip displacement	~ 2 s	1.9 ± 0.2 mN

Table S3. Representative examples of related light-driven with film and bilayered architecture.

Section S11 – FEM simulations of a 6-NO_2-BIPS/PMMA cantilever with internal asymmetric structure

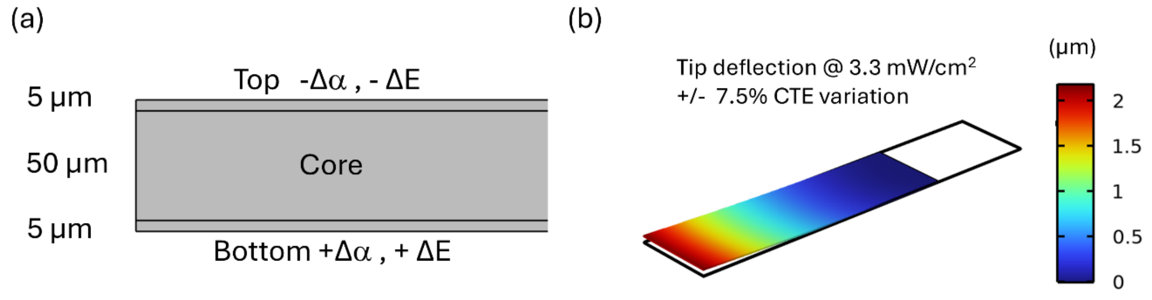

Figure S11. FEM model used to study internal through-thickness asymmetry in 6-NO_2-BIPS/PMMA. (a) Side-view scheme of the three-layer geometry used in the simulations, consisting of 5/50/5 µm top/core/bottom regions. The outer layers are assigned equal and opposite variations relative to the core. (b) Representative simulated deformation for a case with ±7.5% CTE variation, shown as the cantilever tip deflection map under 3.3 mW cm^{-2} illumination (bending visually amplified × 100).

CTE asymmetry, $\pm\Delta\alpha/\alpha_{core}$ (%) \ Young's modulus asymmetry, $\pm\Delta E/E_{core}$ (%)	**0**	**±5**	**±10**	**±15**	**±20**
**0**	0.04	0.04	0.04	0.04	0.04
**±2.5**	0.71	0.71	0.71	0.70	0.70
**±5**	1.38	1.38	1.38	1.38	1.38
**±7.5**	2.05	2.05	2.05	2.04	2.04
**±10**	2.72	2.72	2.72	2.72	2.71

Table S4. Three-layer PMMA simulation matrix. Simulated cantilever tip deflection (µm) for the three-layer 6-NO_2-BIPS/PMMA model (5/50/5 µm top/core/bottom). CTE variations are expressed as

$\pm\Delta\alpha/\alpha_{core}$ for the coefficient of thermal expansion (rows) and Young's modulus variations are expressed as $\pm\Delta E/E_{core}$ layer. The 0/0 entry corresponds to the symmetric reference case.

Section S12. Estimation of the photothermal conversion efficiency

The photothermal conversion efficiency, defined as the ratio of the generated thermal power to the input optical power,[S16] is estimated by analyzing the post-illumination thermal relaxation, namely the cooling region of the temperature profile of our system (Figure 6). The temperature decay after switch-off of the 405 nm excitation, given the small temperature excursion, can be fitted by a single exponential,

$$\Delta T(t) = A_{\Delta T\ Cool}\ e^{-t/\tau_{\Delta TCool}},$$

where ΔT is the difference between the system temperature and room temperature, $\tau_{\Delta TCool}$ is the thermal relaxation time and $A_{\Delta T\ Cool}$ is the temperature amplitude (Figure S12). The fit yields the values, $\tau_{\Delta TCool}$ =3.78 s and $A_{\Delta T\ Cool}$ = 1.55 K. The overall thermal loss coefficient is obtained from the relaxation time through:

$$US_{\text{exch}} = \frac{C_{\text{th}}}{\tau_{\Delta T,\text{cool}}},$$

where S_{exch} is the total exposed heat-exchange area of the cantilever and the total thermal capacitance C_{th} of the cantilever is calculated from the geometry and material parameters used in Section S9, by summing the PMMA and rGO contributions. This procedure yields an effective heat-transfer coefficient, $U \sim 12$ W m^{-2} K^{-1}. The photothermal conversion efficiency can be estimated as:

$$\eta_{\text{th}} = \frac{US_{\text{exch}}\ A_{\Delta T\ Cool}}{P_{\text{in}}},$$

where P_{in}= 1.75 mW is the incident optical power on the illuminated cantilever, with 405 nm illumination at 7 mW cm^{-2}. Since in our experiments the sample reflectance is not independently

measured, the obtained value should be regarded as a lower-limit estimate of the photothermal conversion efficiency. This procedure yields $\eta_{\mathrm{th}} \sim 55\%$.

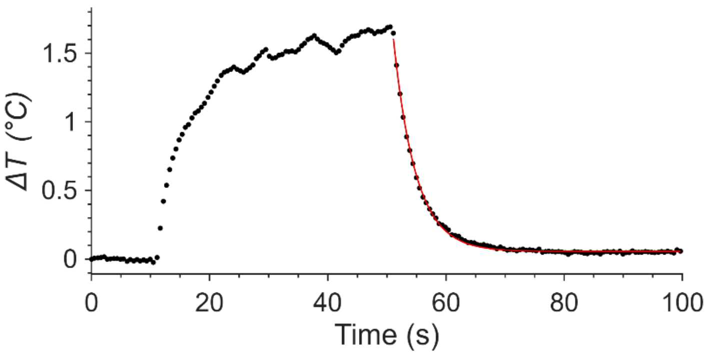

Figure S12. Single-exponential fit of the post-illumination cooling profile of temperature.

References

(S1) Wang, J. F.; Li, P. H.; Tian, X. B.; Shi, S. Q.; Tam, L. Molecular Investigation on Temperature-Dependent Mechanical Properties of PMMA/CNT Nanocomposite. *Eng. Fract. Mech.* **2023**, *293*, 109705.

(S2) Huh, S. H.; Choi, S.-H.; Ju, H.-M.; Kim, D.-H. Properties of Interlayer Thermal Expansion of 6-Layered Reduced Graphene Oxide. *Journal of the Korean Physical Society* **2014**, *64* (4), 615–618.

(S3) Richeton, J.; Ahzi, S.; Vecchio, K. S.; Jiang, F. C.; Makradi, A. Modeling and Validation of the Large Deformation Inelastic Response of Amorphous Polymers over a Wide Range of Temperatures and Strain Rates. *Int. J. Solids Struct.* **2007**, *44* (24), 7938–7954.

(S4) Renteria, J. D.; Ramirez, S.; Malekpour, H.; Alonso, B.; Centeno, A.; Zurutuza, A.; Cocemasov, A. I.; Nika, D. L.; Balandin, A. A. Strongly Anisotropic Thermal Conductivity of Free-Standing Reduced Graphene Oxide Films Annealed at High Temperature. *Adv. Funct. Mater.* **2015**, *25* (29), 4664–4672.

(S5) Han, B.; Zhang, Y.; Zhu, L.; Li, Y.; Ma, Z.; Liu, Y.; Zhang, X.; Cao, X.; Chen, Q.; Qiu, C.; Sun, H. Plasmonic-Assisted Graphene Oxide Artificial Muscles. *Adv. Mater.* **2019**, *31* (5), 1806386.

(S6) Chang, J.; Toga, K. B.; Paulsen, J. D.; Menon, N.; Russell, T. P. Thickness Dependence of the Young's Modulus of Polymer Thin Films. *Macromolecules* **2018**, *51* (17), 6764–6770.

(S7) Athanassiou, A.; Kalyva, M.; Lakiotaki, K.; Georgiou, S.; Fotakis, C. All-Optical Reversible Actuation of Photochromic-Polymer Microsystems. *Adv. Mater.* **2005**, *17* (8), 988–992.

(S8) Zhang, X.; Yu, Z.; Wang, C.; Zarrouk, D.; Seo, J.-W. T.; Cheng, J. C.; Buchan, A. D.; Takei, K.; Zhao, Y.; Ager, J. W.; Zhang, J.; Hettick, M.; Hersam, M. C.; Pisano, A. P.; Fearing, R. S.;

Javey, A. Photoactuators and Motors Based on Carbon Nanotubes with Selective Chirality Distributions. *Nat. Commun.* **2014**, *5* (1), 2983.

(S9) Wang, S.; Gao, Y.; Wei, A.; Xiao, P.; Liang, Y.; Lu, W.; Chen, C.; Zhang, C.; Yang, G.; Yao, H.; Chen, T. Asymmetric Elastoplasticity of Stacked Graphene Assembly Actualizes Programmable Untethered Soft Robotics. *Nat. Commun.* **2020**, *11* (1), 4359.

(S10) Niu, D.; Jiang, W.; Liu, H.; Zhao, T.; Lei, B.; Li, Y.; Yin, L.; Shi, Y.; Chen, B.; Lu, B. Reversible Bending Behaviors of Photomechanical Soft Actuators Based on Graphene Nanocomposites. *Sci. Rep.* **2016**, *6* (1), 27366.

(S11) Lim, H.; Park, T.; Na, J.; Park, C.; Kim, B.; Kim, E. Construction of a Photothermal Venus Flytrap from Conductive Polymer Bimorphs. *NPG Asia Mater.* **2017**, *9* (7).

(S12) Tang, R.; Sang, W.; Wu, Y.; Zhu, C.; Liu, J. Multi-Wavelength Light Drivable Oscillatory Actuator on Graphene-Based Bilayer Film. *Macromol. Mater. Eng.* **2017**, *302* (2), 1600384.

(S13) Wang, X.; Jiao, N.; Tung, S.; Liu, L. Photoresponsive Graphene Composite Bilayer Actuator for Soft Robots. *ACS Appl. Mater. Interfaces* **2019**, *11* (33), 30290–30299.

(S14) Li, C.; Xue, Y.; Han, M.; Palmer, L. C.; Rogers, J. A.; Huang, Y.; Stupp, S. I. Synergistic Photoactuation of Bilayered Spiropyran Hydrogels for Predictable Origami-like Shape Change. *Matter* **2021**, *4* (4), 1377–1390.

(S15) Liman, G.; Mutluturk, E.; Demirel, G. Light- and Solvent-Responsive Bilayer Hydrogel Actuators with Reversible Bending Behaviors. *ACS Materials Au* **2024**, *4* (4), 385–392

(S16) Wang, Y.; Li, M.; Chang, J.-K.; Aurelio, D.; Li, W.; Kim, B. J.; Kim, J. H.; Liscidini, M.; Rogers, J. A.; Omenetto, F. G. Light-Activated Shape Morphing and Light-Tracking Materials

Using Biopolymer-Based Programmable Photonic Nanostructures. *Nat. Commun.* **2021**, *12* (1), 1651.
